# Effects of extreme cyclic loading on the cushioning performance of human heel pads under engineering test condition

**DOI:** 10.3389/fbioe.2023.1229976

**Published:** 2023-10-20

**Authors:** Zhihui Qian, Zhiqiang Zhuang, Xiangyu Liu, Haotian Bai, Lei Ren, Luquan Ren

**Affiliations:** ^1^ Key Laboratory of Bionic Engineering, Ministry of Education, Jilin University, Changchun, Jilin, China; ^2^ Orthopedic Medical Center, The Second Hospital of Jilin University, Changchun, China; ^3^ School of Mechanical, Aerospace and Civil Engineering, University of Manchester, Manchester, United Kingdom

**Keywords:** heel pad, cyclic loading, cushioning performance, dynamic mechanical properties, finite element simulations

## Abstract

Human heel pads commonly undergo cyclic loading during daily activities. Low cyclic loadings such as daily human walking tend to have less effect on the mechanical properties of heel pads. However, the impact of cyclic loading on cushion performance, a vital biomechanical property of heel pads, under engineering test condition remains unexplored. Herein, dynamic mechanical measurements and finite element (FE) simulations were employed to explore this phenomenon. It was found that the wavy collagen fibers in the heel pad will be straightened under cycle compression loading, which resulted in increased stiffness of the heel pad. The stiffness of the heel pads demonstrated an inclination to escalate over a span of 50,000 loading cycles, consequently resulting in a corresponding increase in peak impact force over the same loading cycles. Sustained cyclic loading has the potential to result in the fracturing of the straightened collagen fibers, this collagen breakage may diminish the stiffness of the heel pad, leading to a reduction in peak impact force. This work enhances understanding of the biomechanical functions of human heel pad and may provide potential inspirations for the innovative development of healthcare devices for foot complex.

## Introduction

The human heel pad is the part of the body that initially touches the ground when walking and is divided into five layers from outside to inside: the cuticle, epidermis, dermis, superficial small compartment, and deep large compartment. The fibrous diaphragm is encased in fat to form a deep large compartment (Blechschmidt, 1982; Jahss et al., 1992; Buschmann et al., 1995; Wang et al., 2011), which undergoes a large deformation when the heel pad is subjected to a load to provide a good cushioning performance to accommodate the harsh mechanical environment (Jahss et al., 1992; Rome, 1998).

The heel pad, as a biological tissue in the human body subjected to severe mechanical action, possesses good energy-absorbing properties (Bennett and Ker, 1990), especially its cushioning performance is more outstanding (Kinoshita, 1996). Cushioning performance and cushioning properties are synonymous terms; both denote the capacity of materials to absorb and disperse impact energy when subjected to external forces. The broader notion of energy-absorbing property encompasses various aspects including cushioning performance, damping behavior, and more. This property signifies a material’s ability to absorb and dissipate energy during impact or vibration exposure. Notably, superior energy absorption attributes directly translate to enhanced cushioning performance (Ganesh et al., 2022; Tan et al., 2023). Nonetheless, the viscosity of a material exerts a notable influence on its energy absorption properties (Huang et al., 2021; Saed et al., 2021). When subjected to an impact load, materials with higher viscosity tend to absorb more impact energy, thus yielding heightened cushioning performance. The biomechanical properties of the heel pad have been characterized in the relevant literature, and it was found that the heel pad exhibited hyperelastic mechanical characteristics during compression and that materials with this mechanical characteristic undergo a large deformation under compressive loading (Miller-Young et al., 2002; Grigoriadis et al., 2017), allowing the foot to decelerate over a certain distance and achieve effective cushioning. The stress relaxation and creep of the heel pad reflect the viscoelastic material properties (Behforootan et al., 2017; Suzuki et al., 2017; Tecse et al., 2023). Under viscous action, the impact energy absorbed by the heel pad is dissipated in the form of viscoelastic creep and heat (De Clercq et al., 1994), and the energy dissipated by the heel pad can reach approximately 35% during compression (Ledoux and Blevins, 2007), this is important for cushioning performance. At the same time, the collagen fiber network of the heel pad and the soft adipose tissue are coupled to each other to provide efficient cushioning and vibration damping (Qian et al., 2010).

Studying the effects of cyclic loading on the biological organization of the human body has been a subject of great interest in recent years (Lillie and Gosline, 2007; Chan et al., 2011; van Haaften et al., 2020). Healthy adults take thousands or tens of thousands of steps a day (Tudor-Locke and Bassett, 2004; Tudor-Locke et al., 2011), and heel pad maintains structural integrity and mechanical stability under low cyclic physiological loadings (De Clercq et al., 1994; Wearing et al., 2014). Under engineering test condition, the effects of cyclic loading on damage to the human heel pad and the resulting biomechanical properties have not been reported to the authors’ knowledge. However, the study of human heel pads subjected to excessive cyclic loadings may be able to indirectly assess what kind of changes in heel pad performance occur as a result of too many consecutive steps taken by humans. In this study, the effect of extreme cyclic loading on the cushioning performance of the human heel pad is investigated experimentally, and the biomechanical mechanism of the effect of cyclic loading on the cushioning performance of the human heel pad is assessed in combination with FE simulations. This work can enhance understanding of the biomechanics of the human heel pad, which is important for the daily protection of the human heel pad.

## Methods

### Ethics statement

This work was approved by the ethical committee of the Second Hospital of Jilin University (No. 2021177).

### Cyclic loading tests

**FIGURE 1:**
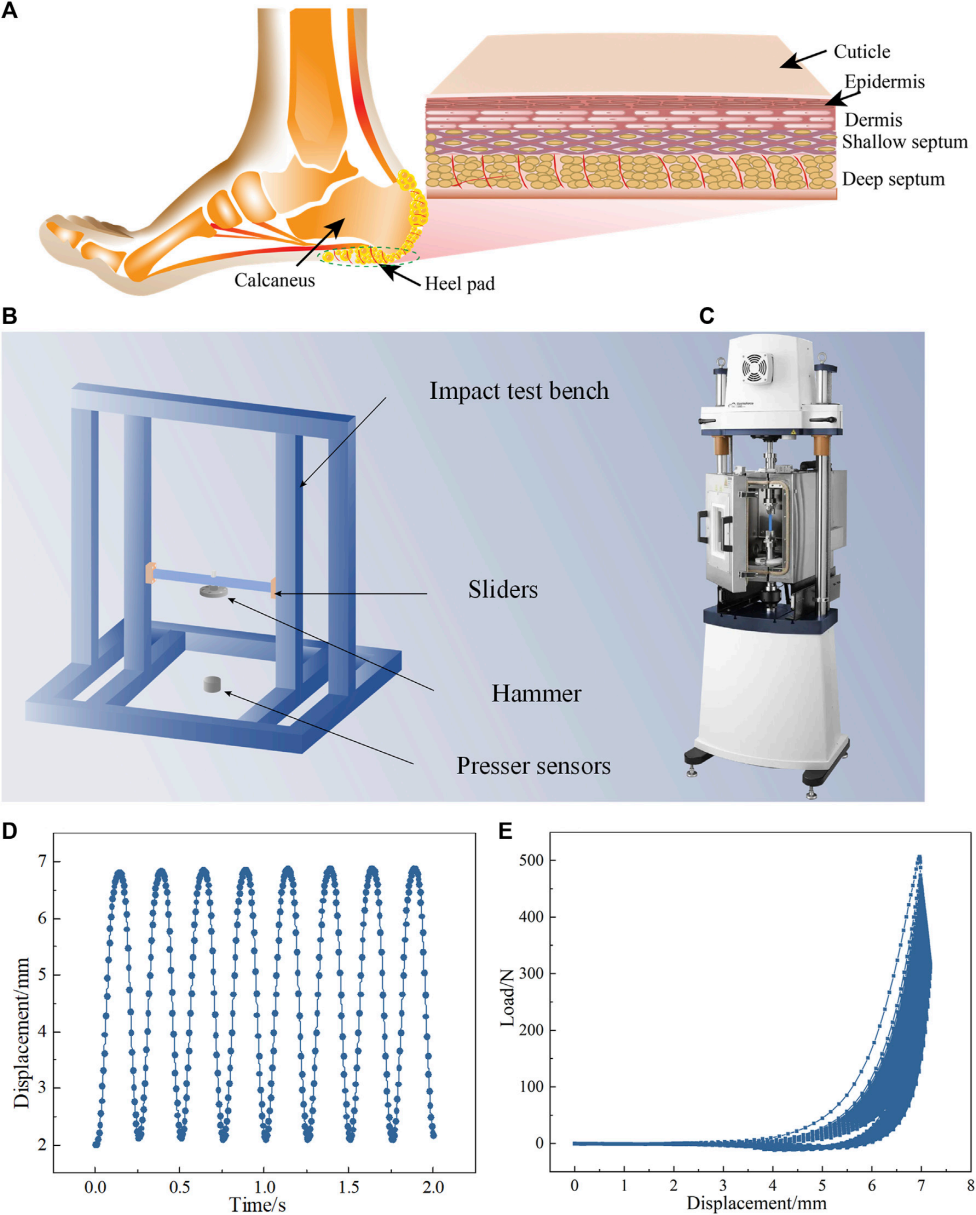
**(A)** A schematic diagram of the structure of the human heel pad, which is located below the heel bone and is divided into five layers from the outside to the inside: the stratum corneum, epidermis, dermis, superficial small compartments and deep large compartments. **(B)** Self-built impact test platform. **(C)** Electro force 3100 tester with dynamic mechanical testing capabilities. **(D)** Cycle loading for displacement control. **(E)** Load‒displacement curves during cycle loading.

Two human heel pad tissue samples were obtained from the Second Hospital of Jilin University and stored on ice at a low temperature of −70°C prior to the test. The heel pads were removed until the test and thawed at room temperature. To avoid water loss and drying of the isolated tissue, we sprayed PBS fluid (Biosharp, China) on the isolated tissue every 10 min during the test, The Electro-force 3100 tester shown in Figure 1C (Waters Technology Co. Ltd., United States) was used to apply cyclic loading to the human heel pad, as shown in Figures 1D, E. The load during the test cannot exceed 70% of the range, i.e., 700N, due to the limitations of the tester. To protect the tester, we set the cyclic compression load to 500N. On the other hand, considering that the number of cycles may be lengthy, we have appropriately reduced the compression load during cycling to avoid excessive damage to the heel pad caused by excessive compression. The control mode of the testing machine during the cyclic loading is displacement control, i.e., the displacement is remained the same during the compression. The compression displacement was 4.88 mm when the compression load was 500 N. Therefore, the compression displacement during cycle loading was 4.88 mm, which ensures a compression load of 500 N for the heel pad, as shown in Figure 1D.

The cushioning performance and dynamic mechanical analysis (DMA) of the heel pad were tested after 10,000 cycles with a total of 60,000 cycles. In this study, the total number of cyclic loading is based on whether or not the surface of the heel pad appears damaged. Until the sixth time, totaling 60,000 cycles, when the surface of the heel pads showed signs of damage, i.e., heel pad fatigue failure, leading us to terminate the test. In order to make the heel pad fatigue damage in a limited time and to make the cyclic loading test completed in a shorter period of time, 10,000 cyclic loading was completed at a frequency of 4 Hz in the cyclic loading test.

### Dynamic mechanical tests

DMA and cushioning performance tests were carried out in time after cyclic loading. The DMA test contains two different frequencies of 1 Hz and 2 Hz, corresponding to the two different states of movement of the human body: walking, and fast running (Osaki et al., 2008; Paradisis et al., 2019). DMA is displacement control with a mean level of −3 mm and an amplitude of 1 mm. Mean level is −3 mm, mean that the tester’s indenter compresses the heel pad by 3 mm. Amplitude is 1 mm, mean that the tester’s indenter moves up and down in that compressed position with an amplitude of 1 mm, so it is 4 mm maximum compression. When the heel pad is impacted, the cushioning performance of the heel pad is mainly realized by the large deformation in the early stage of compression; while the heel pad is stiff in the late stage of compression, which has almost no cushioning effect, so this study only characterizes the dynamic mechanical analysis in the early stage of compression, as shown in the Supplementary Figure S2. Based on this, the maximum compression of the heel pad is 4 mm in the DMA test.

The DMA test indicators mainly include: storage modulus, loss modulus, loss factor (Tan δ), and complex stiffness. The storage modulus is the size of the energy stored due to elastic deformation when the material is deformed, reflecting the size of the elasticity of the material (Jawaid et al., 2013; Saba et al., 2016). The loss modulus, also known as viscous modulus, is the size of the energy lost due to viscous deformation, reflecting the size of the viscosity of the material. Tan δ is the ratio of the loss modulus to the storage modulus and reflects the ratio of the viscoelasticity of the material (Yeni et al., 2004). When the storage modulus is much larger than the loss modulus, the material undergoes mainly elastic deformation, that is, the material is solid, while when the loss modulus is much larger than the storage modulus, the material undergoes mainly viscous deformation, that is, the material is in a liquid state. Complex stiffness represents the ability of material to resist deformation under dynamic loading (Shin and Lee, 2020).

### Construction of impact test benches

An impact test bench is shown in Figure 1B and equipped with a 2.5 kg drop hammer, 10,000 N pressure sensors (Shanghai Li heng Sensor Technology Co., Ltd., China) and an NI data acquisition card (National Instruments, Inc. United States) with a 20 k acquisition frequency to collect the impact load. The drop hammer impact height is 10.3 cm, this is because the impact of drop hammer on the heel pad at this height is comparable to the impact on the sole of the foot when an adult walking. NI DAQ Express software is used to record the impact load data. After each cyclic loading test, an impact test is performed on the heel pad to assess the effect of cyclic loading on its cushioning performance.

In this study, peak impact force and the pressure‒time integral are used as indicators of the cushioning performance (Fan et al., 2022; Li et al., 2022). The peak impact force is the first force when the heel pad is impacted by the drop hammer. Based on the time‒load curve, we calculated the peak force‒time integral at each impact, i.e., the area constituting the region between the force-time curve and the horizontal axis, which represents the impulse of the heel pad. A larger pressure‒time integral indicates that the external load is doing more work on the foot (Vasarhelyi et al., 2006), dissipating more energy and causing more damage to the heel pad. Furthermore, normalized stress-strain curves for heel pads are used and compared with the references. The normalized stress-strain curve is based on the stress-strain curve, it is obtained by dividing the value of stress at each point by its maximum stress, while the strain remains no change.

### Finite element analysis

**FIGURE 2:**
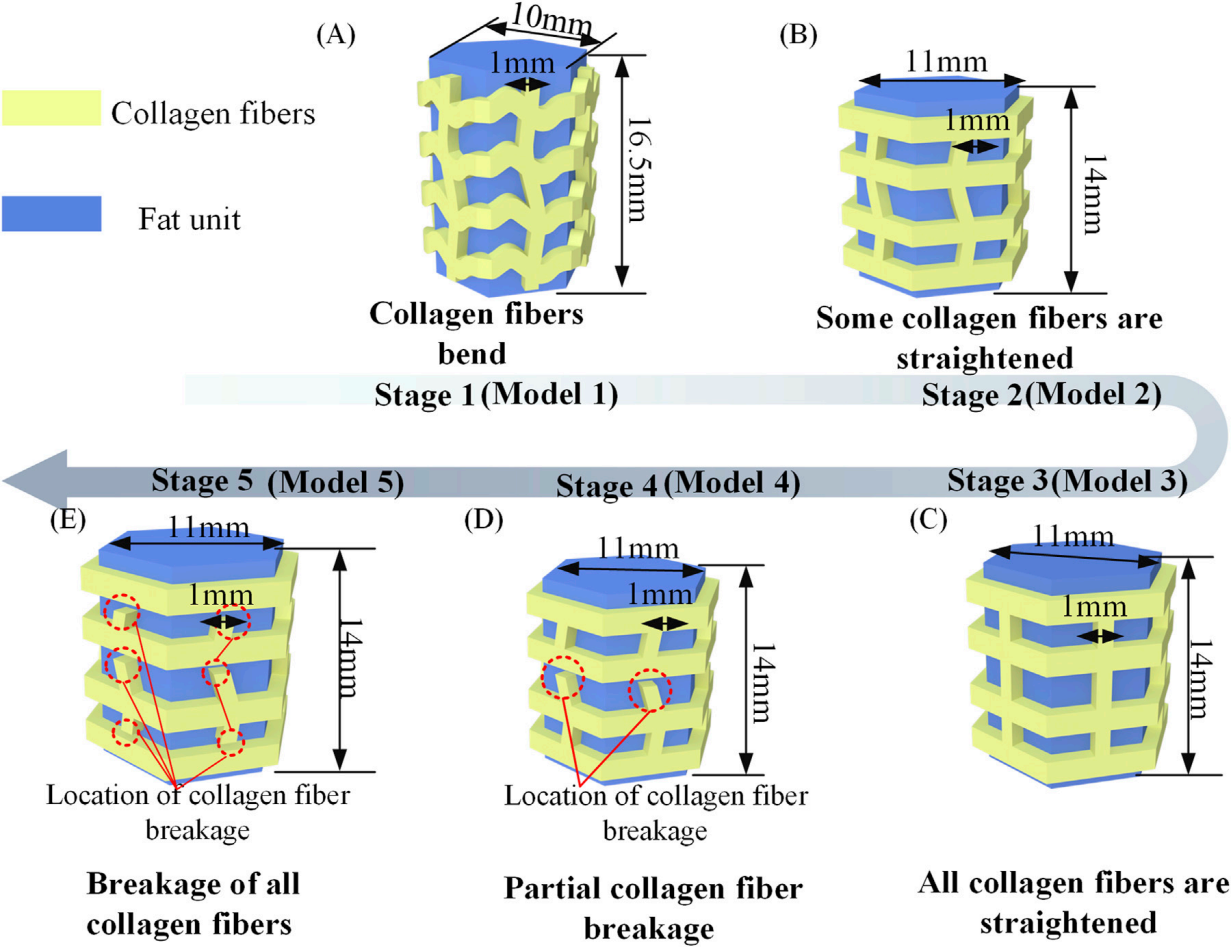
**(A)** Stage 1, Collagen fibers bend. The structure simulates a compartment unit in a heel pad before it is subject to cyclic loading. **(B)** Stage 2, Some collagen fibers are straightened. The structure simulates a compartment unit in a heel pad after some cyclic loading. **(C)** Stage 3, All collagen fibers are straightened. **(D)** Stage 4, Partial collagen fiber breakage. The structure simulates a compartment unit in a heel pad after a large amount of cyclic loading. **(E)** Stage 5, Breakage of all collagen fibers.

Abaqus software (Simulia, Providence, United States) is used to perform FE simulations of the heel pad compartment unit. The FE model was based on the compartment units in human heel pads, which are independent of each other. The compartment unit consists of collagen fibers and fat unit, in which the collagen fibers are wrapped around the surface of the fat unit, and the collagen fibers have a wavy shape and the fat unit has a columnar structure (Blechschmidt, 1982; Rome, 1998). Based on the existing studies on heel pad compartments, since collagen fibers are the main component that constitutes the septum, and the thickness of the septum is usually around 1 mm (Natali et al., 2012), the thickness of collagen fibers in this study is 1 mm. Based on the structural dimensions of the human heel pad, as shown in Supplementary Material, the height of the FE model in this study are 16.5 mm. When the compartment unit does not undergo cyclic loading, the collagen fibers have a curved wavy structure with a fat unit height of 16.5 mm and a square hexagonal cross section, with the structural dimensions shown in Figure 2A.

FE simulation used a static simulation in this study, the solver used is Abaqus/Standard. Since the human heel pads have the mechanical properties of nonlinearity and large deformation during compression, we turned on the geometric nonlinearity in analysis step section, as shown in Supplementary Figure S7. As too high number of cycles tends to cause the computations to not converge, the compartment unit was compressed four times in 1s, i.e., the compression frequency was 4 Hz, settings related to the cyclic loading are shown in Supplementary Figure S8. After applying compression displacement of 4.88 mm to the FE model, the load on the compartment unit is 2N. Cyclic compression loading can cause irreversible structural changes and result in a reduction in the height of the material (Joshi et al., 2006; Van Den Broek et al., 2012). On this basis, a compartment unit model was developed after experiencing cyclic loading to investigate the changes in mechanical properties of the compartment unit after some of the collagen fibers were straightened, as shown in Figure 2B. This model was used to simulate the compartment unit in the heel pad after a certain cyclic compression load, which has the same volume as that of Figure 2A. Next, all collagen fibers are modeled with straightened structural dimensions, which were used to simulate the compartment unit after undergoing a further cyclic loading, as shown in Figure 2C. Further, we cut the collagen fibers where the stress is concentrated, the model is shown in Figures 2D, E. The effect of collagen fiber fracture on the mechanical properties of the compartment unit is further investigated.

Material coefficients for collagen fibers using second-order simplified polynomial strain energy potentials (Ou et al., 2018), The parameters are as follows, C10 (N/mm^2^): 8.42E-03, C20 (N/mm^2^): 7.78E-03, D1 (mm^2^/N): 2.39, D2 (mm^2^/N): 0. The elastic modulus of the fat unit is 0.03 MPa, and the Poisson’s ratio is 0.49 (Ou et al., 2018). The mesh information and the contact settings are the same for all models in this study. After mesh sensitivity analysis, the mesh type of the fat unit is a 3D hexahedron with a mesh size of 0.8 mm; the mesh unit of the collagen fibers is a 3D tetrahedron with a mesh size of 1 mm; and the collagen fibers are attached to the fat unit, as shown in Supplementary Figure S5. The contact between the upper and lower surfaces of the compartment unit and the rigid body is defined as a frictionless surface-to-surface contact, and the load is applied at the reference point of the rigid body, as shown in Supplementary Figure S4. Based on the load-displacement curve of the compartment unit in the FE simulation, the stiffness of the initial compression was obtained, i.e., the slope of the straight line, as shown in Supplementary Figure S11. More information on these models is in the Supplementary Material.

## Results

### Dynamic mechanics experimental results

**FIGURE 3:**
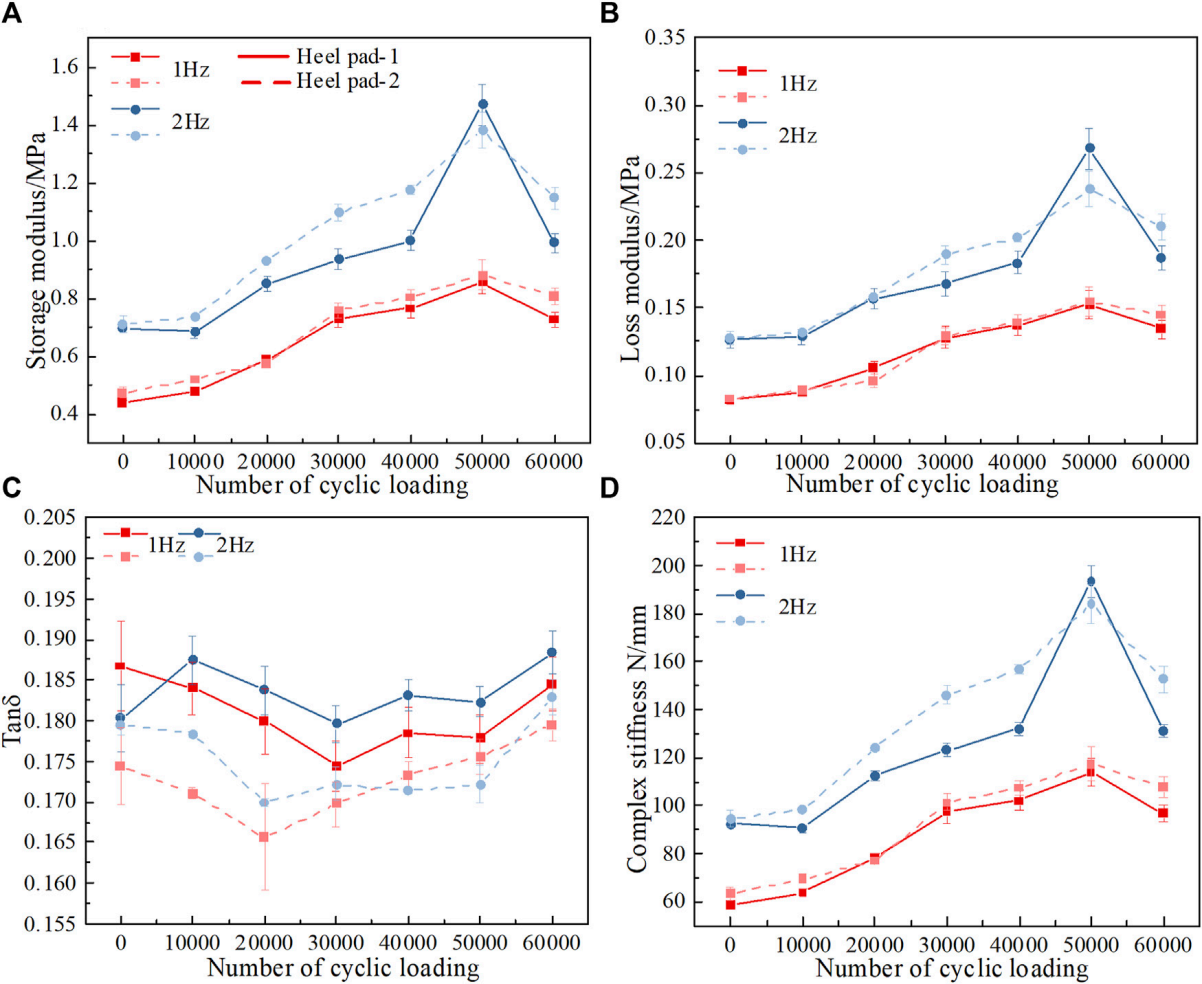
Dynamic mechanics experimental results. **(A)** Storage modulus after different numbers of cycle loadings. **(B)** Loss modulus after different numbers of cycle loadings. **(C)** Tan δ after different numbers of cycle loadings. **(D)** Complex stiffness after different numbers of cycle loadings.

Prior to the cushion test, a dynamic mechanical characterization of the heel pad was performed, focusing on the effect of cyclic loading on the properties of the heel pad, such as the storage modulus, loss modulus, and complex stiffness. The test results show that the storage modulus and the loss modulus increase and then decrease as the number of cyclic loadings increases at two different frequencies: 1 Hz and 2 Hz, as shown in Figures 3A, B. It is worth noting that as the frequency increases, the storage modulus and loss modulus also gradually increase, i.e., storage modulus 2 Hz > storage modulus 1 Hz and loss modulus 2 Hz > loss modulus 1 Hz. The 2 Hz and 1 Hz values correspond to two different gait patterns for healthy adults: running, and walking, respectively. As the walking speed increases, the impact force on the heel pad gradually increases; during this process, the modulus of loss increases to dissipate some of the impact energy and adapt to the different gait patterns.

The Tanδ values of the heel pad at the two different frequencies varied in the range of 0.17–0.2, with an overall trend of decreasing and then increasing at the two different frequencies, as shown in Figure 3C. Since 0<tan δ< 1, the heel pad shows viscoelastic solid material properties.

**FIGURE 4:**
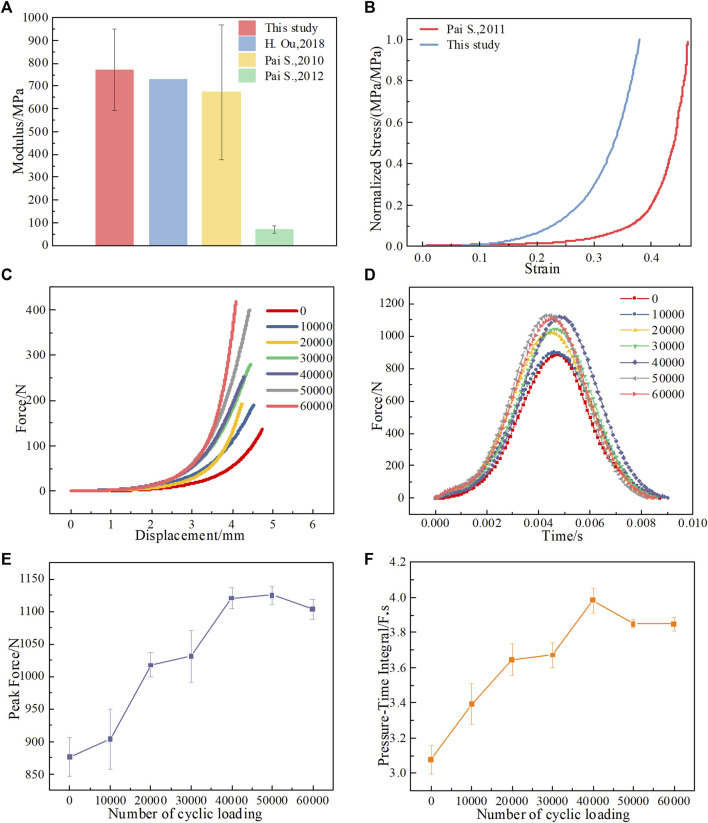
Results of cushioning experiments. **(A)** Modulus of heel pads, modulus is the slope of the straight line after the point of the stress-strain curve inflection., compared with references (Pai and Ledoux, 2010; Pai and Ledoux, 2012; Ou et al., 2018). **(B)** Comparison of normalized stress-strain curve of heel pads under compressive loading among studies (Pai and Ledoux, 2011). **(C)** Load‒displacement curves after different numbers of loading cycles. **(D)** Impact force‒time curves after different numbers of cycle loadings. **(E)** Peak impact force after different numbers of cycle loadings. **(F)** Pressure‒time integral after different numbers of cycle loadings.

Since the tissue stiffness has an important influence on its mechanical properties (Allan et al., 2022), the complex stiffness under different cyclic loads is investigated in this study. The complex stiffness of the heel pad increases gradually with the number of loading cycles and reaches its maximum when the number of loading cycles reaches 50,000, i.e., the heel pad reaches its stiffest state, while the composite stiffness decreases after more than 50,000 cycles, i.e., the heel pad softens once again, as shown in Figure 3D. The trend in stiffness can also be seen in the load‒displacement curve in Figure 4C.

### Cushioning performance

We characterized the mechanical properties of the heel pad before they were subjected to cyclic loading and compared them with the results of the tests in the references. Consistent with the test results of heel pads in the references, the curves are the same in trend. The normalized stress-strain curve measured in this study is slightly higher than that measured in the literature (Pai and Ledoux, 2011), i.e., the modulus of the heel pads measured in this study is higher than the results in the references (Pai and Ledoux, 2010), as shown in Figures 4A, B. In this study, it is believed that the samples used for the compression tests in the references were heel pads that did not contain a skin layer and were cut into pieces, which might resulted in a more soft material property of the heel pads (Jahss et al., 1992). However, the heel pads used in this study are not cut into pieces, and the heel pads contain a skin layer. Since the skin layer tends to limit deformation of the heel pad, the modulus of the heel pads may increase (Rome, 1998; Hsu et al., 2007). In addition, reference (Pai and Ledoux, 2012) provides the shear modulus of the heel pad, which is much lower compared to the compression modulus of the heel pad, as shown in Figure 4A, this may be due to the different mechanical properties of the heel pad in the shear and compression directions.

The test results show that the peak impact force gradually increases with the number of loading cycles, and the peak impact force gradually increases with the number of loading cycles. After more than 50,000 loading cycles, the peak impact force decreases again, as shown in Figures 4D, E.

As shown in Figure 4F, the test results show that as the number of cycle loadings increases, the pressure‒time integral value increases, i.e., the work done by the heel pad gradually increases, and the cushioning performance gradually deteriorates, but the value decreases when the number of cyclic loadings reaches 50,000.

Although the peak impact force and pressure‒time integral decrease after more than 50,000 cycles of loading, the heel pad is in a structurally damaged state due to continuous cyclic loading and does not reflect the cushioning performance in its full structural state. That is, the reduction in the peak impact force of the heel pad under sustained cyclic loading comes at the expense of its structural damage.

### FE analysis of heel pad compartment unit

**FIGURE 5:**
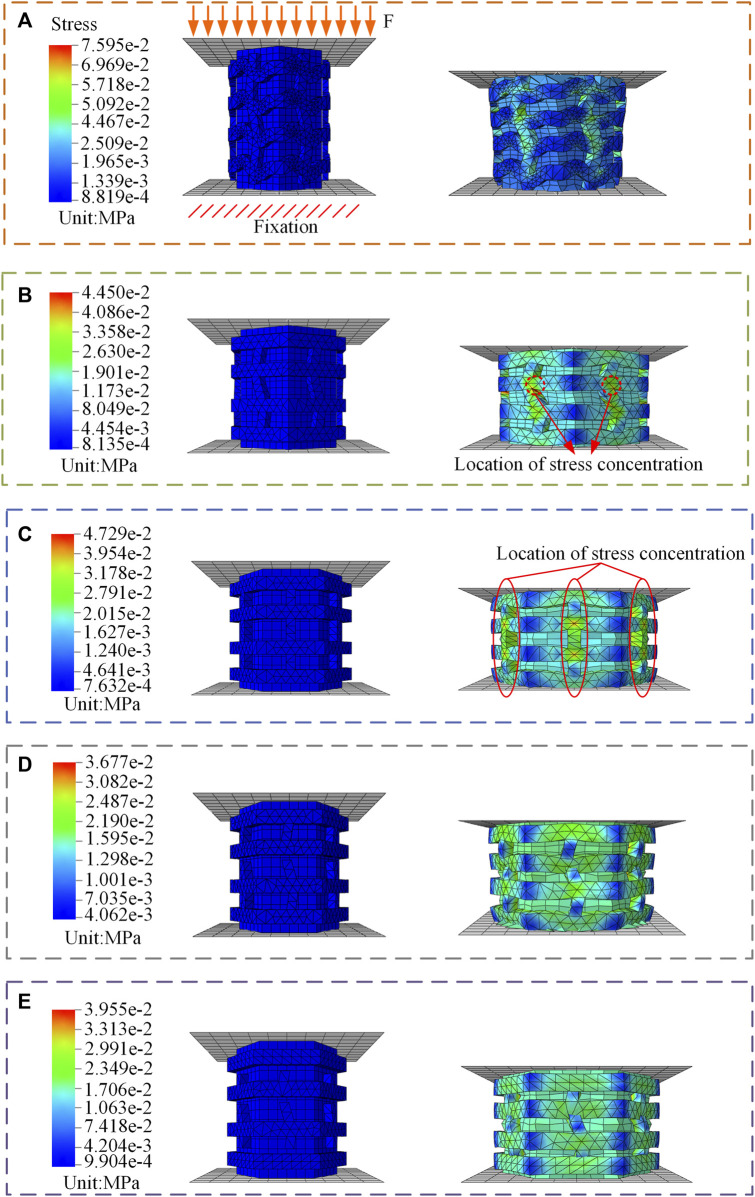
FE simulation results. **(A)** Simulation results of the bending collagen fiber compartment unit. **(B)** Simulation results of part of the collagen fibers being straightened in the compartment unit. **(C)** Simulation results of a compartment unit with all collagen fibers straightened. **(D)** Simulation results of the compartment unit with partial collagen fiber breakage. **(E)** Simulation results of the compartment unit with all collagen fibers broken.

**FIGURE 6:**
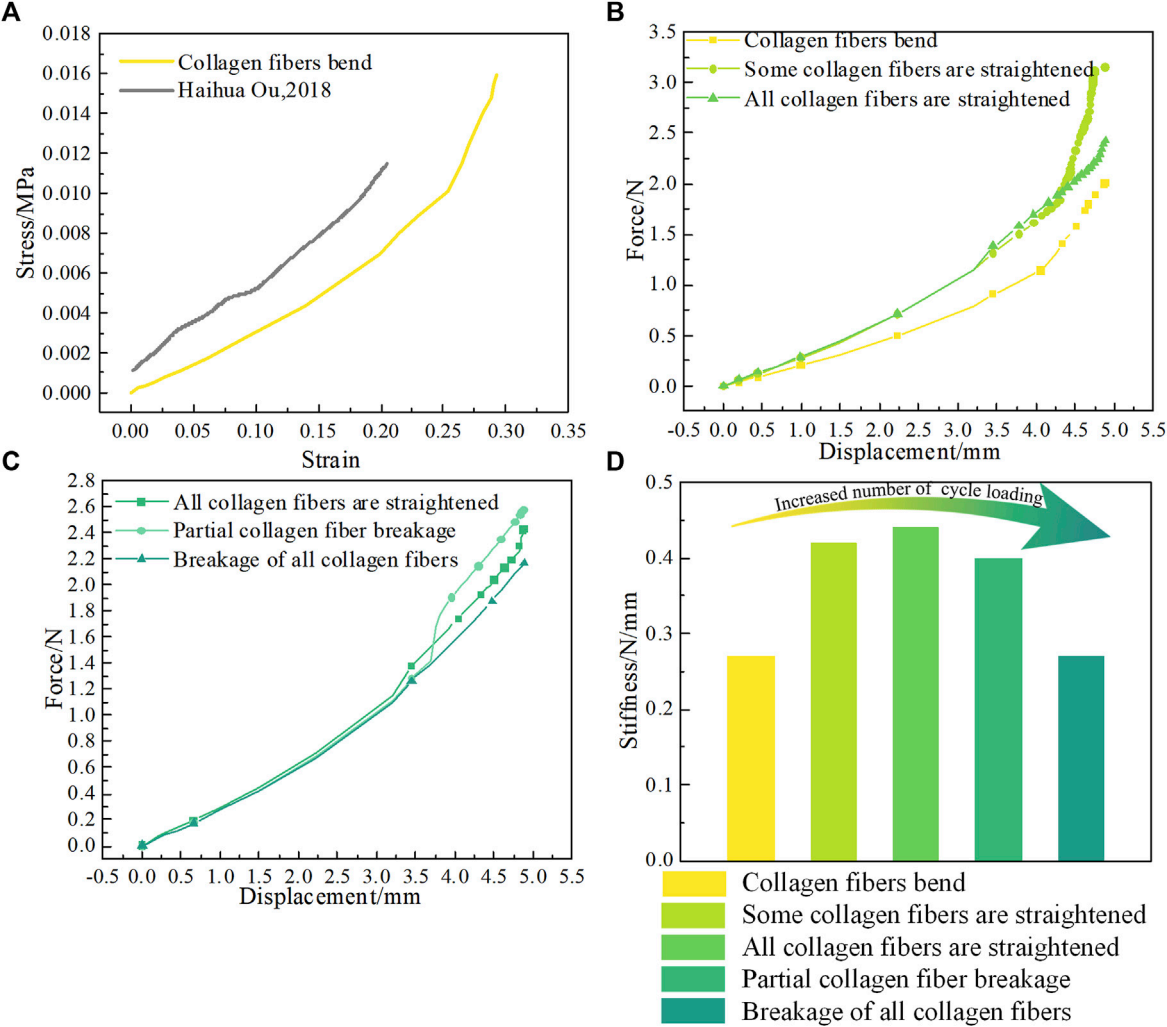
**(A)** Stress‒strain curve of the bending collagen fiber compartment unit under compressive load. **(B)** Load‒displacement curves for different numbers of collagen fiber bends. **(C)** Load‒displacement curves at different collagen fiber breakage numbers. **(D)** Stiffness of compartment units with different collagen fiber structures.

In stage 1, when the compartment unit does not undergo cyclic loading, the collagen fibers have a curved wavy structure, the mechanical properties of the compartment unit under compressive loading are very similar to those in the literature, as shown in Figure 6A. After the compartment unit under cycle loading was compressed by 4.88 mm, some of the collagen fibers were straightened, as shown in Supplementary Movie 3 and Figure 5A, and the compartment unit with bent collagen fibers had the lowest stiffness, as shown in Figure 6B. In stage 2, a compartment unit model was developed to investigate the changes in mechanical properties of the compartment unit after some of the collagen fibers were straightened, as shown in Figure 2B. In the compartment unit after 4.88 mm compression, the stress concentration phenomenon occurred on the collagen fibers, as shown in Figure 5B; at this point, some of the collagen fibers are straightened, and the stiffness of the compartment unit increases, as shown in Figure 6B. Next, In stage 3, all collagen fibers are modeled with straightened structural dimensions. Under the compression load, the stress concentration on the collagen fibers is more pronounced, which indicates that damage is most likely to occur there under compressive loading, as shown in Figure 5C, and the stiffness of the compartment unit continues to increase as the collagen fibers are all straightened, as shown in Figure 6B. The effect of collagen fiber fracture on the mechanical properties of the compartment unit is further investigated in this study, as shown in Figure 5D. In stage 4, after partial collagen fiber fracture, the stress concentration phenomenon on the collagen fiber is reduced, as shown in Figure 5D, and the stiffness of the compartment unit is reduced due to the collagen fiber fracture, as shown in Figure 6C. In stage 5, When all the collagen fibers are broken, the stress concentration phenomenon is no longer obvious, as shown in Figure 4E, and the stiffness of the compartment unit is lowest at this time, as shown in Figure 6C.

In conclusion, after the collagen fiber bent compartment unit experienced cycle loading, the collagen fiber was first gradually straightened, resulting in an increase in the compartment unit stiffness. As the collagen fiber was pulled off, and the compartment unit stiffness decreased instead, as shown in Figure 6D. The FE simulation results explaining why the stiffness of the heel pad first increases and then decreases after experiencing cyclic loading. It was analyzed as a result of structural changes in the collagen fibers.

## Discussion

In this study, the relationship between heel pad cyclic loading and cushioning performance is investigated under engineering test condition, and the biomechanical mechanism behind the relationship is revealed by FE simulation. The heel pad compartment unit is formed by a fibrous membrane wrapped around a fat unit (Blechschmidt, 1982; Buschmann et al., 1995), and the fibrous membrane is mainly composed of collagen fibers in a spiral shape (Buschmann et al., 1995), as shown in Figure 1A. Cyclic loading usually causes degradation of the stiffness of biological tissues by destroying their structure (Mirzaali et al., 2020; Firminger and Edwards, 2021; Zitnay et al., 2021), which in turn causes the degradation of biomechanical function. A thorough understanding of the heel pad compartment structure is necessary to study its biomechanical mechanism.

In the cyclic loading tests of this study, low-number of cycle loadings are not enough to significantly affect the mechanical properties of heel pads (De Clercq et al., 1994; Wearing et al., 2014), in order to assess the mechanical properties of heel pads after being subjected to excessive cyclic loadings, we appropriately extend each cyclic loading to 10,000 cycles, for a total of 60,000 cycles in 6 times, and investigated the cushioning performance of heel pads under extreme experimental conditions. For humans under normal physiological conditions, this is a study of the effects of an excessive number of consecutive walking steps on the cushioning properties and dynamic mechanical properties of the heel pad. The experimental results of this study show that heel pads harden under non-physiological conditions as the number of cyclic loading increases, leading to a decrease in the cushioning performance of the heel pads. For humans under physiological conditions, this result may predict that excessive walking steps can also lead to a decrease in cushioning performance due to heel pad hardening. It has been shown that different compression frequencies have very little effect on the mechanical property performance of heel pads (Miller-Young et al., 2002; Ledoux and Blevins, 2007). Therefore, 4Hz, which is slightly higher than the frequency of human movement, was chosen to complete 10,000 cyclic loadings in this study. The frequency is necessary, if the frequency is too low, the cycle loading every cycle of 10,000 times will take too long, it is difficult to ensure that the test is completed in a short time.

The DMA results show that the storage modulus of the heel pad tends to increase and then decrease with the number of loading cycles, as shown in Figure 3A. We believe that this phenomenon is due to the wavy collagen fibers being gradually stretched and straightened under compressive loading, with the collagen fibers losing their elasticity as they are straightened (Ker, 1999; Grytz and Meschke, 2009), resulting in an increase in the overall modulus of the heel pad and a greater stiffness. Later, the fracture of the collagen fibers causes the compartments to rupture, and the fat is easily extruded from between the collagen fiber webs, resulting in an overall softening of the heel pad and a reduction in stiffness (Jahss et al., 1992; Rome, 1998). We have verified the above conjecture by means of FE simulation, and we used different structures of collagen fiber wrapped around the same structure of fat unit to simulate the heel pad after different cycles of loading. Heel pads made of straightened collagen fibers have a higher stiffness than wavy collagen fibers, as shown in Figure 6D. The collagen fibers are cut at the location of stress concentration, and the collagen fracture reduced the stiffness of the heel pad and softened the heel pad through FE simulation, as shown in Figure 6D.

The storage modulus of the heel pad increases with the number of loading cycles, the stiffness of the heel pad also increases, the overall deformation of the heel pad decreases when subjected to impact load, and the cushioning performance deteriorates. When the number of cycle loadings reached 60,000, the collagen fiber septum of the heel pad broke down, as shown in Supplementary Figure S1. The collagen fibers of the heel pad break down, resulting in a reduction in the overall storage modulus and stiffness of the heel pad. The overall deformation of the heel pad increases when subjected to impact loading, and the peak impact force decreases, i.e., the reduction of the peak impact force of the heel pad comes at the cost of its structural breakage. The loss modulus of the heel pad tends to increase and then decrease after cycle loading, as shown in Figure 3B, although its viscosity increases and some of the impact energy is lost, as its storage modulus is much larger than the loss modulus, it resists the impact load mainly through elastic deformation during the impact process, i.e., the stiffness affected by the heel pad structure is the main factor affecting the cushioning performance of the heel pad.

We also found an interesting phenomenon through our experiments when heel pad at different frequencies, storage modulus 2 Hz > storage modulus 1 Hz and loss modulus 2 Hz > loss modulus 1 Hz, as shown in Figures 3A, B. The 2 Hz and 1 Hz values correspond to two different gait patterns for healthy adults: fast running, and walking, respectively. As the walking speed increases, the impact force on the heel pad gradually increases; during this process, the modulus of loss increases to dissipate some of the impact energy to protect the foot as much as possible.

In this study, an important rule was found that the cushioning performance of the *in vitro* heel pads deteriorated as the number of cycle loadings increased and that the structure of the heel pads were damaged when the number of cycle loadings reached 60,000. Both cases of *in vitro* heel pads were from healthy adults who were non-athletes, and there may be slight individual differences between them for such identical groups of people, which will be further discussed in future work. This finding may have important implications for the daily protection of the heel pad and to avoid damage to the internal structure of the heel pad.

The number of *in vitro* heel pads used for testing in this work is limited. In future work, it is recommended that the number of *in vitro* heel pads be increased to obtain more refined results. Future work may also include optimization of the heel pad compartment simulation to create a model that more closely matches the human heel pad compartment unit to obtain more reliable simulation results.

## Conclusion

In this study, under engineering test condition, we characterized the effects of cyclic loading on the cushion performance of a human heel pad by building an impact test bench and TA test machine. Based on FE analysis, the biomechanical mechanism of the effect of cyclic loading on cushioning performance was revealed. The cyclic loading affect the structural characteristics of the collagen fibers in the fibrous membrane of the heel pad, and the structural characteristics of the collagen fibers determine the cushioning performance of the heel pad. This study can enhance understanding of the biomechanical mechanism of the human heel pad and may provide new inspiration for the protection of foot sports such as walking and running. In order to prevent the cushioning of the heel pad from deteriorating and avoid damage to the heel pad due to excessive continuous walking, controlling the number of consecutive walking steps may be beneficial.

## Data Availability

The raw data supporting the conclusion of this article will be made available by the authors, without undue reservation.
