# Supplementary material for: Effects of extreme cyclic loading on the cushioning performance of human heel pads under engineering test condition
Source: Front Bioeng Biotechnol. 2023 Oct 20;11:1229976. doi: 10.3389/fbioe.2023.1229976 (PMC10623005; doi:10.3389/fbioe.2023.1229976)
Supplement: Supplementary file 3 [file DataSheet1.docx]

Supplementary Material

**Effects of Cyclic Loading on the Cushion Performance of Human Heel Pads**

**Zhihui Qian^1,*^, Zhiqiang Zhuang^1^, Xiangyu Liu^1^, Haotian Bai^3^, Lei Ren^1,2,*^, Luquan Ren^1^**

*** Correspondence:**

zhqian@jlu.edu.cn

Lei Ren

[lei.ren@manchester.ac.uk](mailto:lei.ren@manchester.ac.uk)


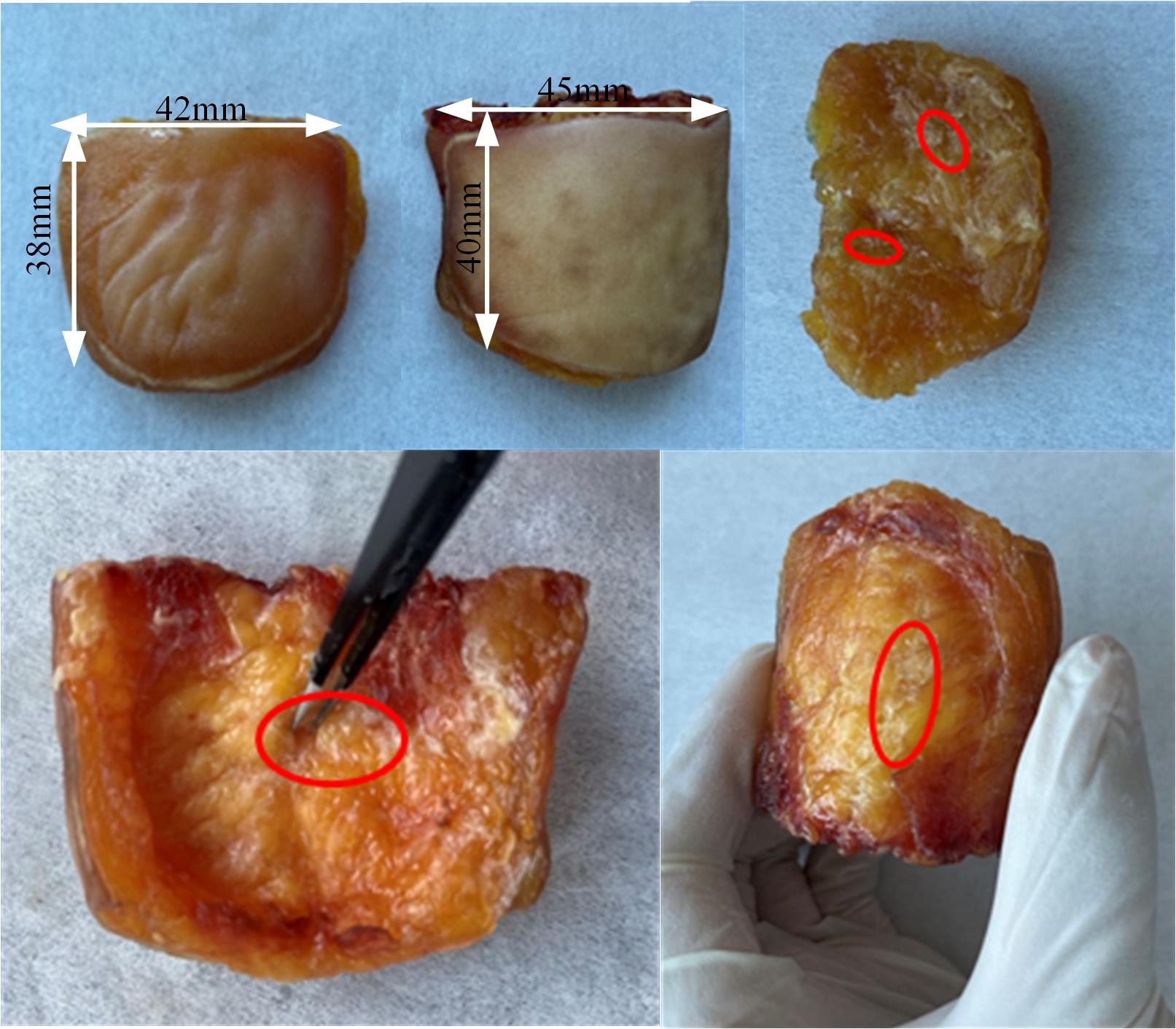


**Supplementary Figure 1.** Two cases of human heel pads used in the test.

To avoid structural damage to the heel pad that could affect the test results, we did not remove the skin and other tissues from the heel pad. After 60,000 cycles of loading, the fibrous membrane on the surface of the heel pad is significantly damaged, the rupture location is marked by the red circle.


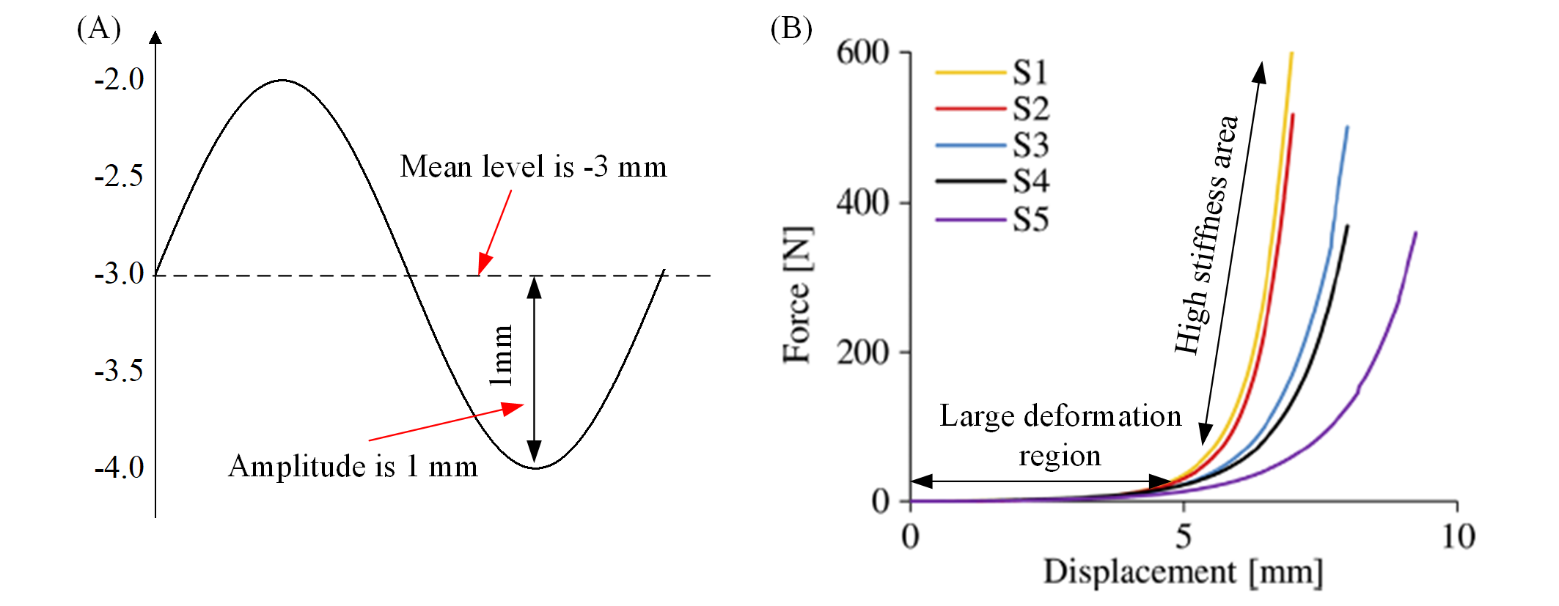


**Supplementary Figure 2**. (A) Schematic diagram of DMA test parameter setting. (B) The quasi-static compressive force–displacement curves for all 5 heel pad samples (Grigoriadis et al., 2017).

**References:**

Grigoriadis, G., Newell, N., Carpanen, D., Christou, A., Bull, A. M. J., and Masouros, S. D. (2017). Material properties of the heel fat pad across strain rates. *J. Mech. Behav. Biomed. Mater.* 65, 398–407. doi: 10.1016/j.jmbbm.2016.09.003.


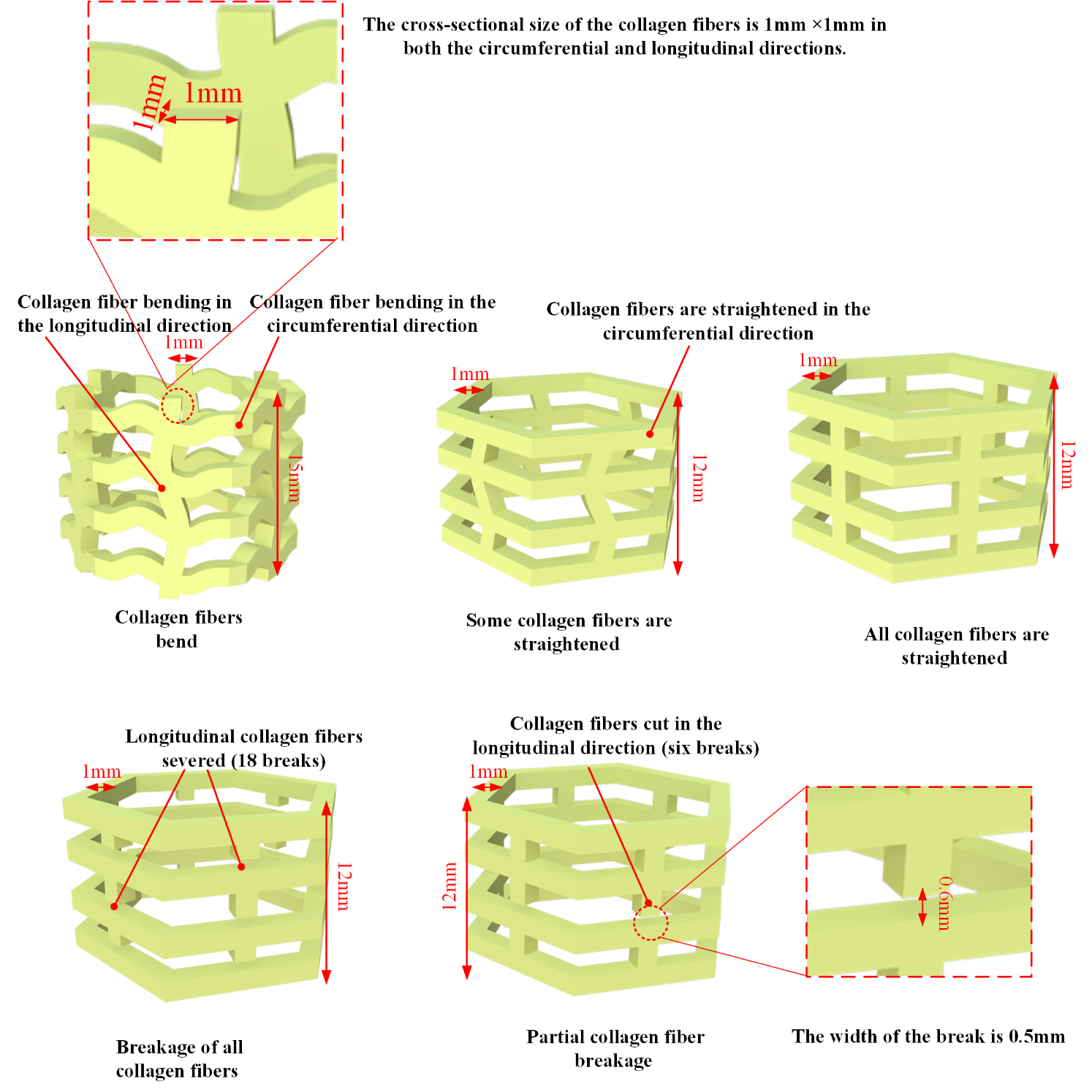


**Supplementary Figure 3.** Some structural details of collagen fibers.


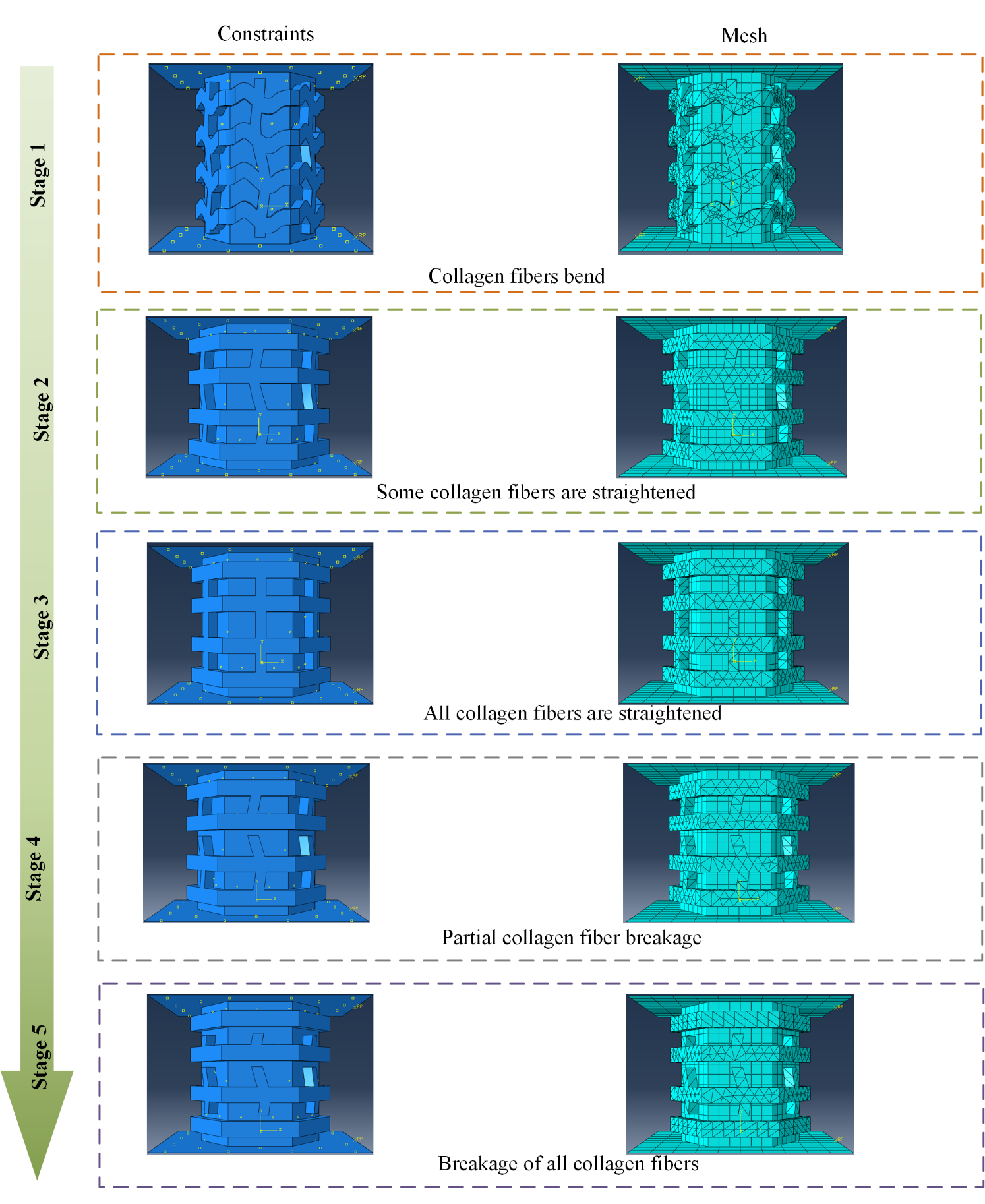


**Supplementary Figure 4.** Constraints and mesh of the model during FE simulation.


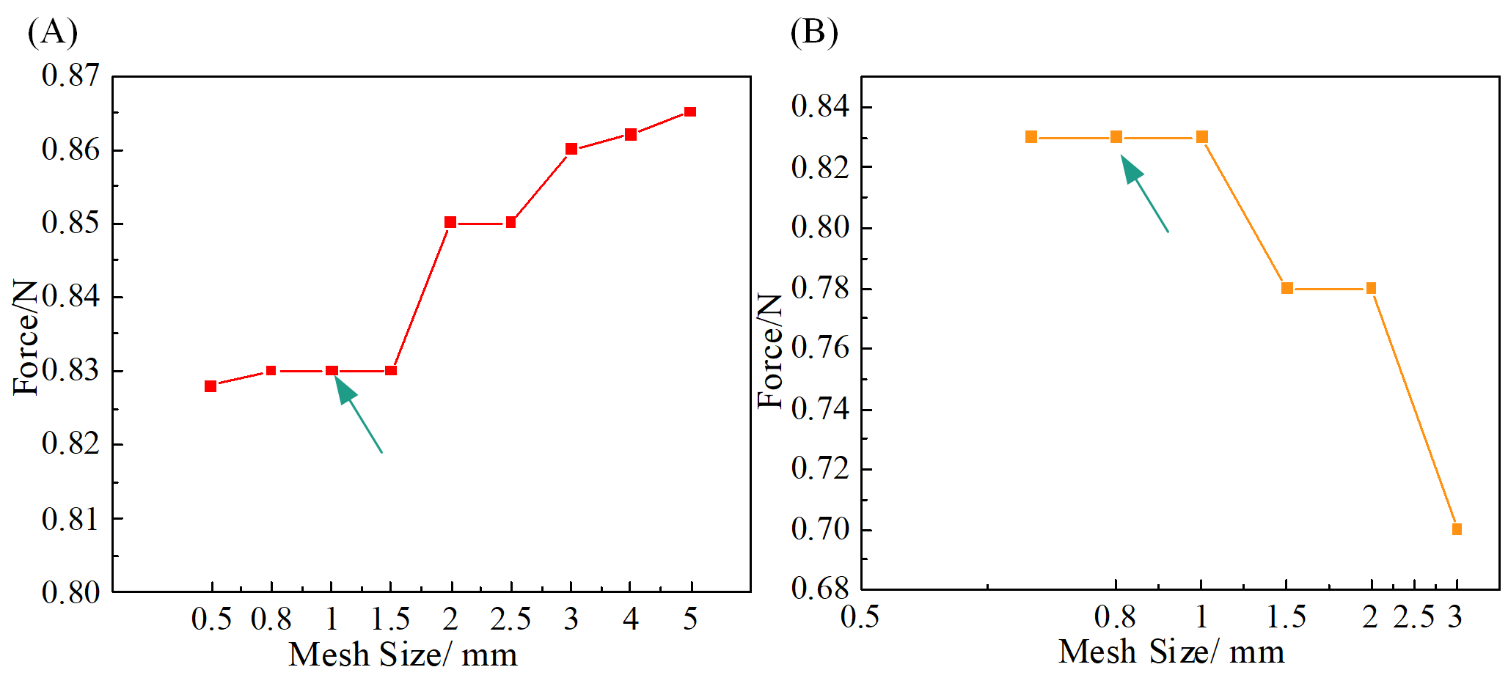


**Supplementary Figure 5**. (A) Mesh sensitivity analysis of collagen fibers. (B) Mesh sensitivity analysis of fat unit.

By mesh sensitivity analysis of the model, a mesh size of 1 mm has been determined for the collagen fibers and a mesh size of 0.8 mm for the fat cells, as shown in Supplementary Figure 4.


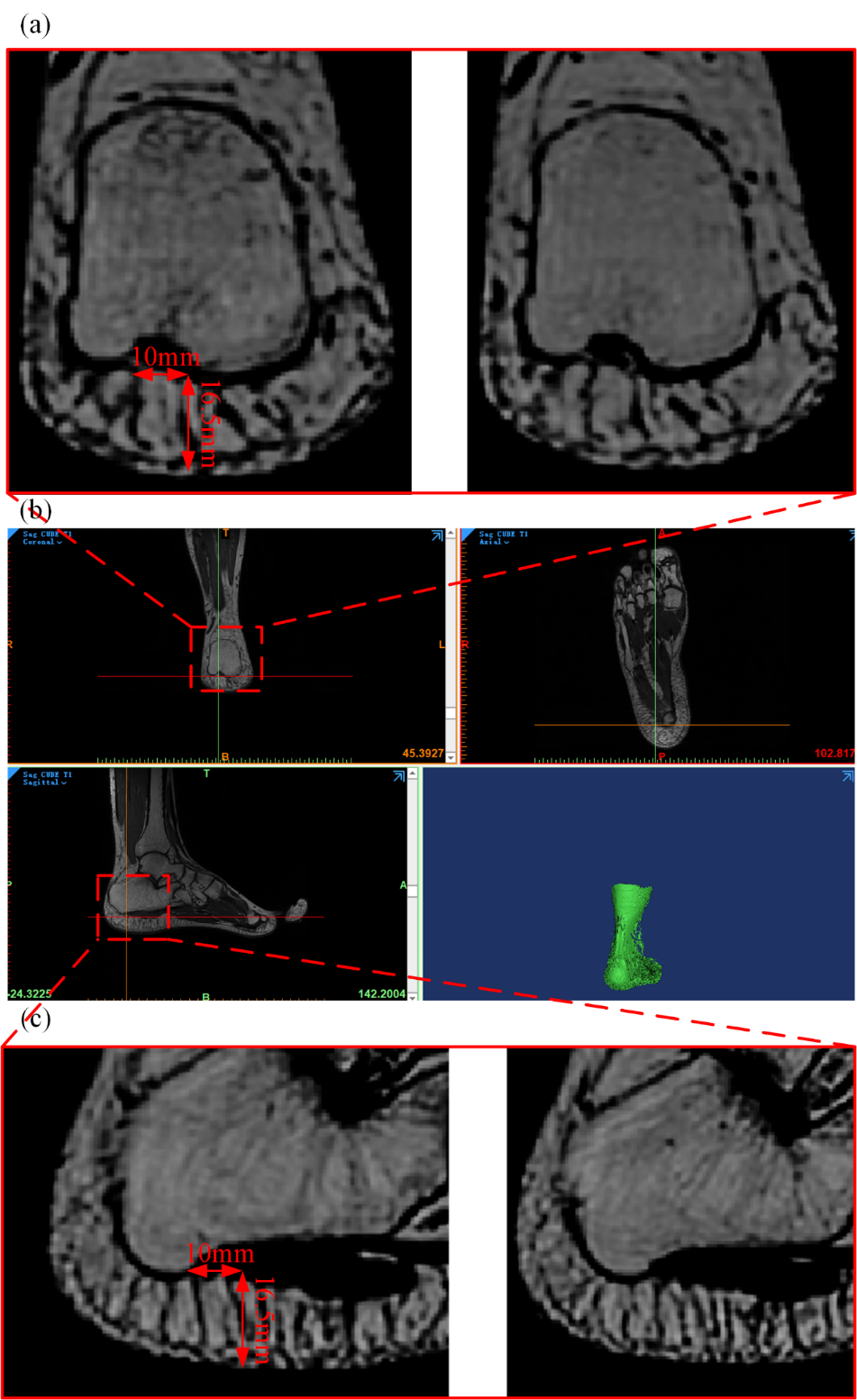


**Supplementary Figure 6**. (B) MRI image of the heel pad.


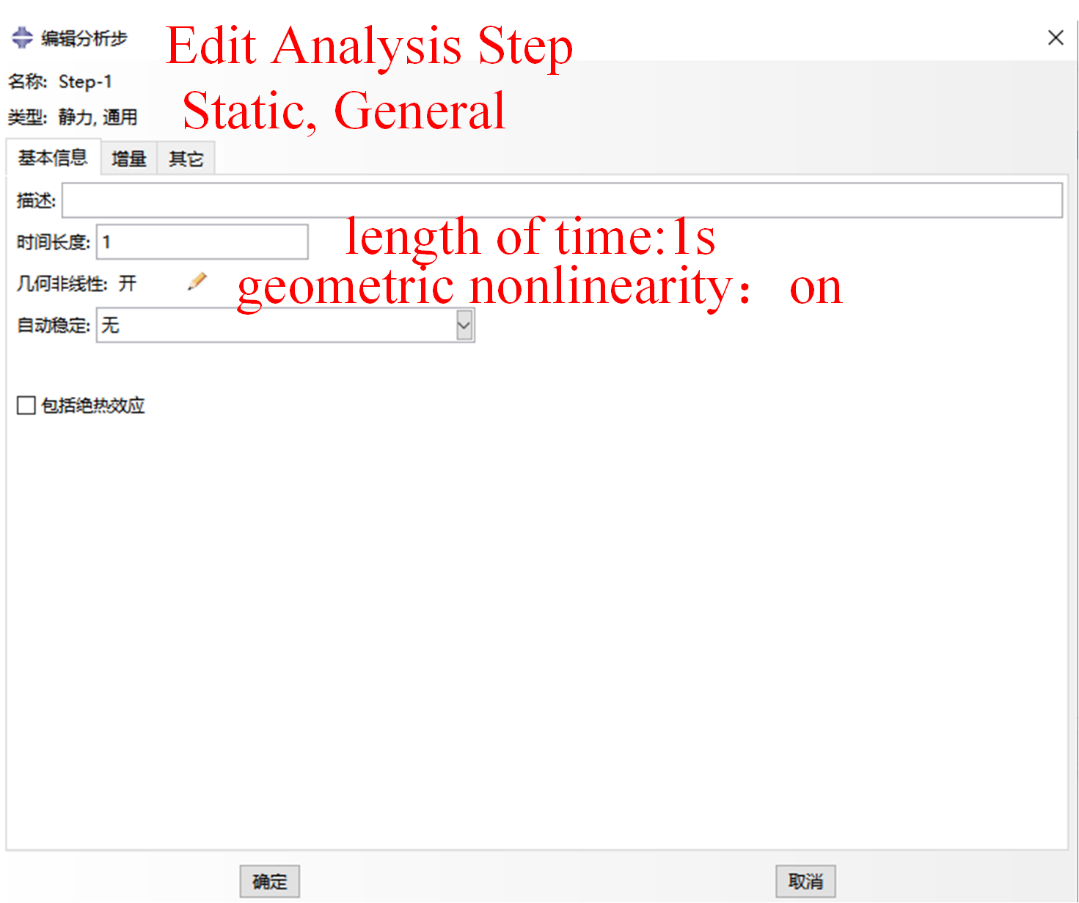


**Supplementary Figure 7.** Setup of the analysis step.


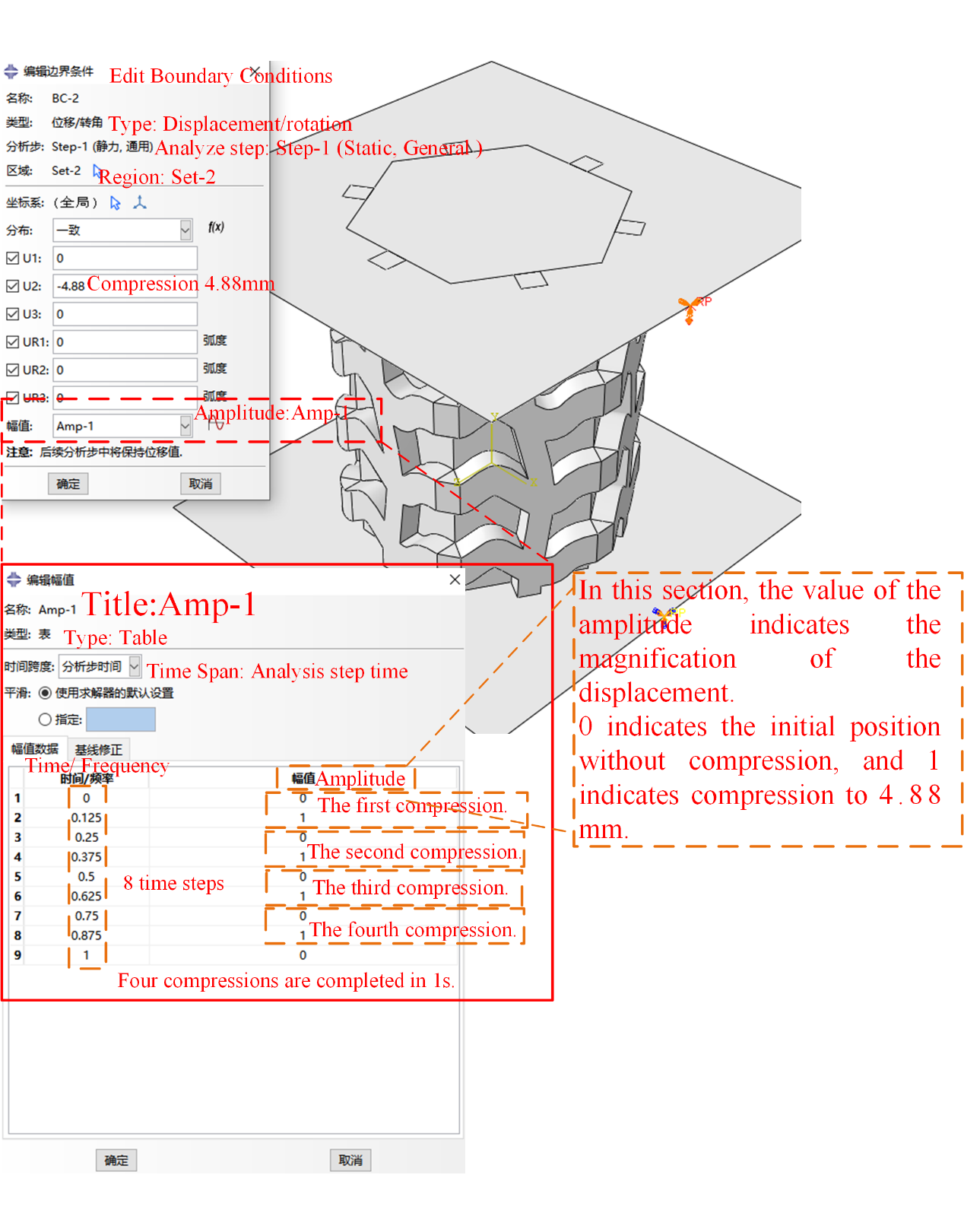


**Supplementary Figure 8.** Setup of Boundary Condition and Amplitude

In this study, the model was compressed four times, which was done under static general section. In the analysis step section, we set the time step to 1, as shown in Supplementary Figure 7, and make the relevant settings of time and amplitude in the Edit Amplitude section of Edit Boundary Condition, as shown in Supplementary Figure 8. The total time was 1, i.e., the compartment unit was repeatedly compressed 4 times, as shown in the supplemental video (FE simulation).


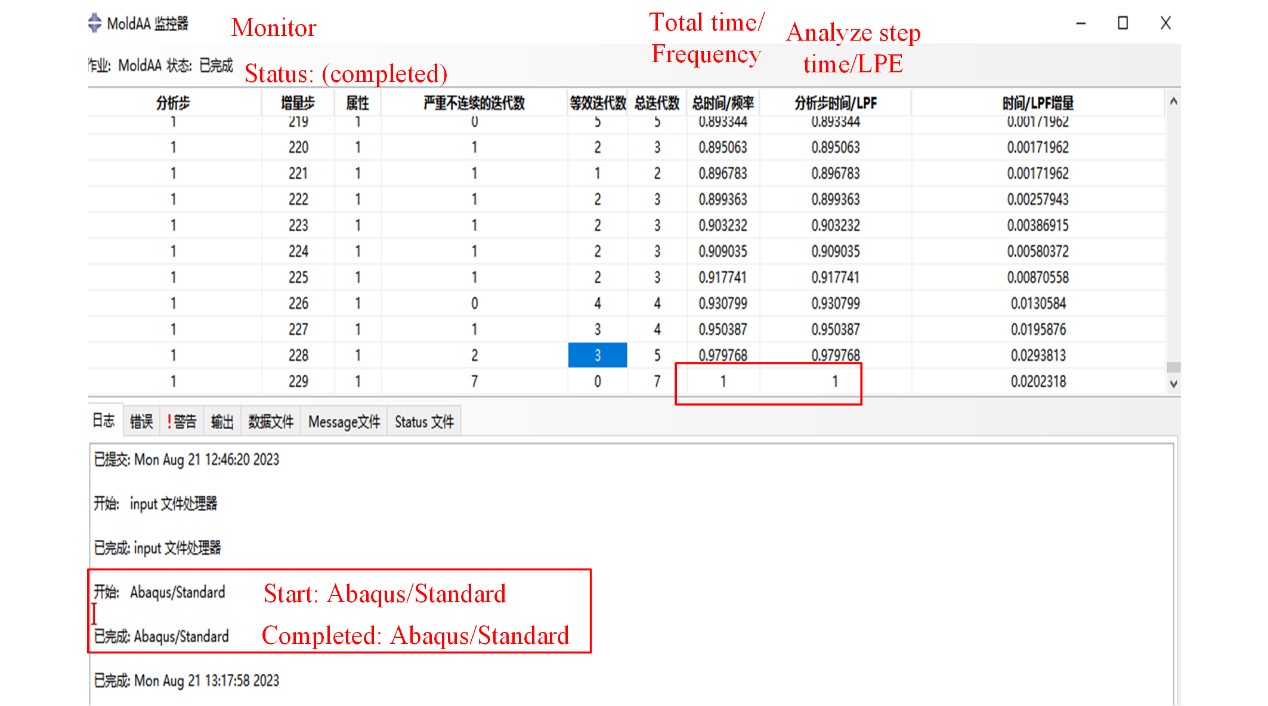


**Supplementary Figure 9.** Monitor for FE emulation


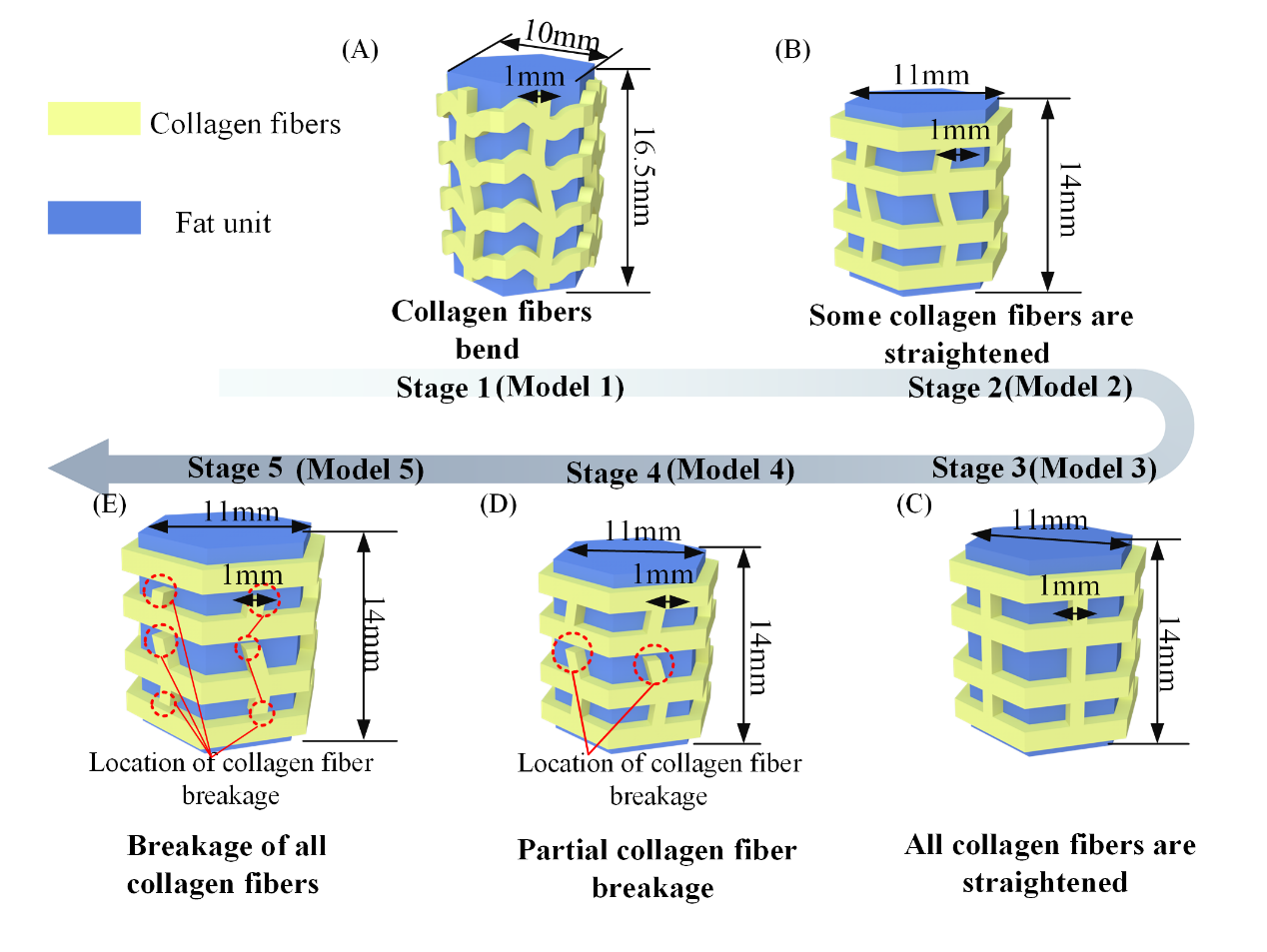


**Supplementary Figure 10.** (A) Stage 1, Collagen fibers bend. The structure simulates a compartment unit in a heel pad before it is subject to cyclic loading. (B) Stage 2, Some collagen fibers are straightened. The structure simulates a compartment unit in a heel pad after some cyclic loading. (C) Stage 3, All collagen fibers are straightened. (D) Stage 4, Partial collagen fiber breakage. The structure simulates a compartment unit in a heel pad after a large amount of cyclic loading. (E) Stage 5, Breakage of all collagen fibers.

In this study, we first built the model in Supplementary Figure 10 (A), mimicking the heel pad without cyclic loading applied, and we applied four cycle loading in the FE simulation. During this process, we observed that the wavy collagen fibers being repeatedly straightened. Due to the tendency of cyclic loading to induce irreversible structural changes (Joshi et al., 2006; Van Den Broek et al., 2012). Based on this, a compartment unit model was developed to investigate the mechanical property alterations after the straightening of some collagen fibers post-cyclic loading, as depicted in Supplementary Figure 10 (B). Subsequently, we created the model in Supplementary Figure 10 (C) to simulate the compartment unit in the heel pad after a certain number of cycles, and we applied four cycle loading on the model in the FE simulation. During the FE simulation, we observed that there was a more pronounced concentration of damage on the collagen fibers, indicating that they are more susceptible to damage under compressive loading. Based on the findings, we then strategically cut the collagen fibers at the regions of concentrated stress, as depicted in Supplementary Figure 10 (D) and (E). By applying FE simulations to the above two models, we investigated the effects caused by excessive cyclic loading on the mechanical properties of heel pads.


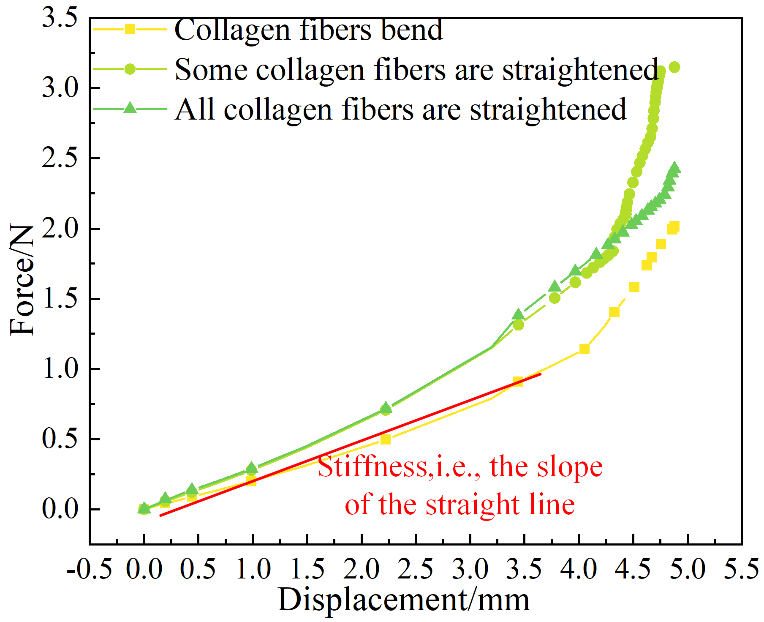


**Supplementary Figure 11.** Calculation of initial stiffness.

Based on the load-displacement curve of the compartment unit in the FE simulation, we obtained the stiffness of the initial compression, i.e., the slope of the straight line. When the heel pad is impacted, the cushioning performance of the heel pad is mainly realized by the large deformation in the early stage of compression; while the heel pad is stiff in the late stage of compression, which has almost no cushioning effect, so this study only characterizes the the stiffness of the initial compression.
